# Sildenafil Ameliorates Gentamicin-Induced Nephrotoxicity in Rats: Role of iNOS and eNOS

**DOI:** 10.1155/2014/489382

**Published:** 2014-07-10

**Authors:** Mohamed A. Morsy, Salwa A. Ibrahim, Entesar F. Amin, Maha Y. Kamel, Rehab A. Rifaai, Magdy K. Hassan

**Affiliations:** ^1^Department of Pharmacology, Faculty of Medicine, Minia University, El-Minia 61511, Egypt; ^2^Department of Histology, Faculty of Medicine, Minia University, El-Minia 61511, Egypt; ^3^Department of Physiology, Faculty of Medicine, Minia University, El-Minia 61511, Egypt

## Abstract

Gentamicin, an aminoglycoside antibiotic, is used for the treatment of serious Gram-negative infections. However, its usefulness is limited by its nephrotoxicity. Sildenafil, a selective phosphodiesterase-5 inhibitor, was reported to prevent or decrease tissue injury. The aim of this study is to evaluate the potential protective effects of sildenafil on gentamicin-induced nephrotoxicity in rats. Male Wistar rats were injected with gentamicin (100 mg/kg/day, i.p.) for 6 days with and without sildenafil. Sildenafil administration resulted in nephroprotective effect in gentamicin-intoxicated rats as it significantly decreased serum creatinine and urea, urinary albumin, and renal malondialdehyde and nitrite/nitrate levels, with a concomitant increase in renal catalase and superoxide dismutase activities compared to gentamicin-treated rats. Moreover, immunohistochemical examination revealed that sildenafil treatment markedly reduced inducible nitric oxide synthase (iNOS) expression, while expression of endothelial nitric oxide synthase (eNOS) was markedly enhanced. The protective effects of sildenafil were verified histopathologically. In conclusion, sildenafil protects rats against gentamicin-induced nephrotoxicity possibly, in part, through its antioxidant activity, inhibition of iNOS expression, and induction of eNOS production.

## 1. Introduction

Gentamicin is an effective aminoglycoside antibiotic against serious Gram-negative bacterial infections. Nevertheless, its widespread clinical use is restricted by its nephrotoxic side effect that occurs in up to 30% of treated patients [1]. The pathogenesis of gentamicin nephrotoxicity involves multiple pathways, including oxidative stress, inflammation, reduced renal blood flow, and increased nitric oxide (NO) level [2, 3]. Several agents have been used, with various degrees of success, to ameliorate or prevent gentamicin nephrotoxicity [4–6].

Sildenafil, a selective phosphodiesterase-5 inhibitor, exerts multiple pharmacological actions that involve increase in intracellular cGMP levels, scavenging of free radicals, and decrease in inflammatory cytokines [7]. Moreover, sildenafil induces iNOS and eNOS expression [8]. These pharmacological multiactions offer the possibility of interacting with various pathological conditions usually through one or more of these actions. For example, sildenafil is now being used to treat not only erectile dysfunction but also pulmonary hypertension. It also may have potential for treating several other conditions, including ischemia/reperfusion injury, myocardial infarction, heart failure, stroke, neurodegenerative diseases, and Raynaud's phenomenon [9]. Therefore, the present study aimed to determine whether sildenafil can protect rat kidney from gentamicin-induced nephrotoxicity and to demonstrate the possible mechanisms.

## 2. Methods

### 2.1. Chemicals

Sildenafil citrate was a generous gift from Eva Pharma (Giza, Egypt). Gentamicin sulfate was a generous gift from Memphis Pharm. & Chemical Ind. (Cairo, Egypt). Antibodies against iNOS and eNOS were purchased from Thermo Fisher Scientific Inc./Lab Vision (Fremont, CA, USA).

### 2.2. Animals and Experimental Design

Male Wistar rats weighing 150–180 g were used after one week for proper acclimatization to the standard housing conditions (25 ± 2°C temperature and 12 h light/dark cycle) and were supplied with standard rodent chow and tap water ad libitum. Procedures involving animals and their care were conducted according to EEC Directive of 1986 (86/609/EEC). Animals were randomly divided into 4 groups of 6–8 animals each. Group (1) served as the control group. Group (2) was treated with sildenafil (5 mg/kg, p.o.) [10] daily for 6 consecutive days and served as positive control. Group (3) was injected with gentamicin (100 mg/kg, i.p.) [11] daily for 6 consecutive days to induce nephrotoxicity. Group (4) was given sildenafil (5 mg/kg, p.o.) 1 h before gentamicin (100 mg/kg, i.p.) daily for 6 consecutive days. The rats were sacrificed 24 h following the last gentamicin injection, blood samples were collected, and serum was separated by centrifugation at 3000 g for 10 min. A longitudinal section from the left kidney was excised from each animal for histological and immunohistochemical examination. The renal cortex of the rest of the kidneys was stored at –80°C and subsequently homogenized in ice-cold phosphate buffer (0.05 M, pH 7.4) for biochemical analysis.

### 2.3. Biochemical Analysis

Serum levels of creatinine, urea (Diamond Diagnostics, Egypt), and urinary albumin (BioSystems, Spain) as well as renal catalase and superoxide dismutase (Biodiagnostic, Egypt) activities were determined according to the manufacturers' guidelines using commercially available kits. Renal cortex malondialdehyde, an index of lipid peroxidation, was estimated as previously described [12] using 1,1,3,3-tetramethoxypropane as standard. Renal cortex total nitrite/nitrate, the stable oxidation end products of nitric oxide, served as an index of nitric oxide level and was measured by reduction of nitrate into nitrite using activated cadmium granules, followed by color development with Griess reagent in acidic medium [13].

### 2.4. Histological and Immunohistochemical Examination

Samples of renal tissue were fixed in 10% buffered formalin, embedded in paraffin wax, sectioned, stained with hematoxylin and eosin, and examined under light microscope. Expressions of iNOS and eNOS were detected immunohistochemically using UltraVision ONE HRP polymer detection system (Thermo Fisher Scientific Inc./Lab Vision, Fremont, CA, USA). Briefly, kidney sections were deparaffinized and rehydrated, and nonspecific binding was blocked by Ultra V block. Sections were incubated overnight at 4°C with diluted primary antibodies (iNOS, 1 : 100 and eNOS, 1 : 50). Consequently, they were incubated for additional 30 min with UltraVision One HRP polymer followed by color development using diaminobenzidine.

### 2.5. Statistical Analysis

Data were expressed as means ± SEM. The differences among treated groups were performed by one-way ANOVA followed by Tukey's test. The difference of mean was considered statistically significant at a value of *P* < 0.05.

## 3. Results

### 3.1. Effects of Sildenafil on Urea, Creatinine, and Albumin Levels

Data of serum urea and creatinine as well as urinary albumin levels, as markers of renal functions, are summarized in Table 1. Concomitant administration of sildenafil with gentamicin significantly decreased gentamicin-induced elevation in urea, creatinine, and albumin levels.

### 3.2. Effects of Sildenafil on Renal Histopathology

**Figure 1:**
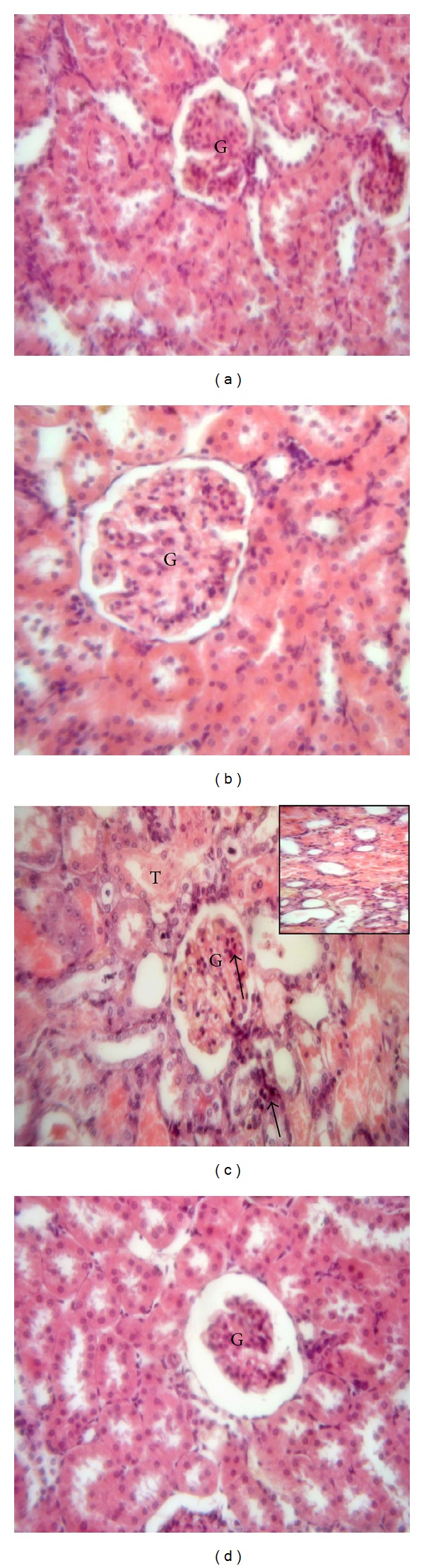
Effect of sildenafil on kidney histopathological picture of gentamicin-induced nephrotoxicity in rats (H&E ×400). A representative photomicrograph of a section in the kidney cortex of (a) and (b) control and sildenafil-treated groups, respectively, showing normal structure of renal glomerulus (G) and renal tubules, (c) gentamicin-treated group showing degeneration and necrobiosis in the epithelial cells lining the renal tubules (T), with apoptotic morphology with pyknotic nuclei in some cells of the renal glomerulus and tubules (arrows) and cystic luminal dilatation in some tubules (inset), and (d) gentamicin- and sildenafil-treated group showing regular epithelial cells lining the tubules with normal morphology of renal cortex.

Histopathological examination revealed that control and sildenafil groups showed normal features of renal glomeruli and cortical tubules (Figures 1(a) and 1(b)). In contrast, gentamicin-treated group showed degeneration and necrobiosis in the epithelial cells lining the renal tubules (Figure 1(c)). Concomitant administration of sildenafil with gentamicin restored the histopathological insult induced by gentamicin, as it showed regular epithelial cells lining the tubules (Figure 1(d)).

### 3.3. Effects of Sildenafil on Renal Malondialdehyde, Nitrite/Nitrate, Catalase, and Superoxide Dismutase

**Figure 2:**
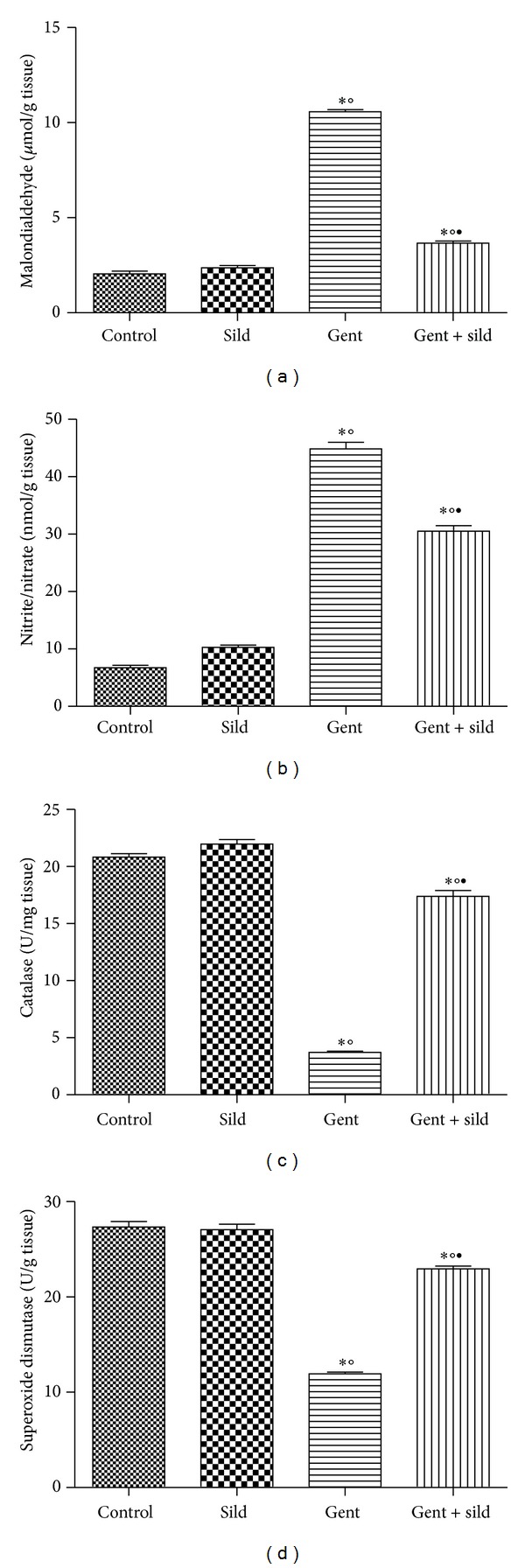
Effect of sildenafil (Sild) on renal malondialdehyde (a), nitrite/nitrate (b), catalase (c), and superoxide dismutase (d) levels of gentamicin- (Gent-)induced nephrotoxicity in rats. Data are mean ± SEM of 6–8 rats. ^∗,°,•^Significantly different from control, sildenafil, and gentamicin groups, respectively, at *P* < 0.05.

Sildenafil treatment significantly decreased the elevation of both malondialdehyde and nitrite/nitrate levels in comparison with gentamicin-intoxicated group (Figures 2(a) and 2(b)). On the other hand, concomitant treatment of sildenafil with gentamicin caused significant increase in renal catalase and superoxide dismutase activities compared with gentamicin-treated group (Figures 2(c) and 2(d)).

### 3.4. Effects of Sildenafil on iNOS and eNOS Expression

**Figure 3:**
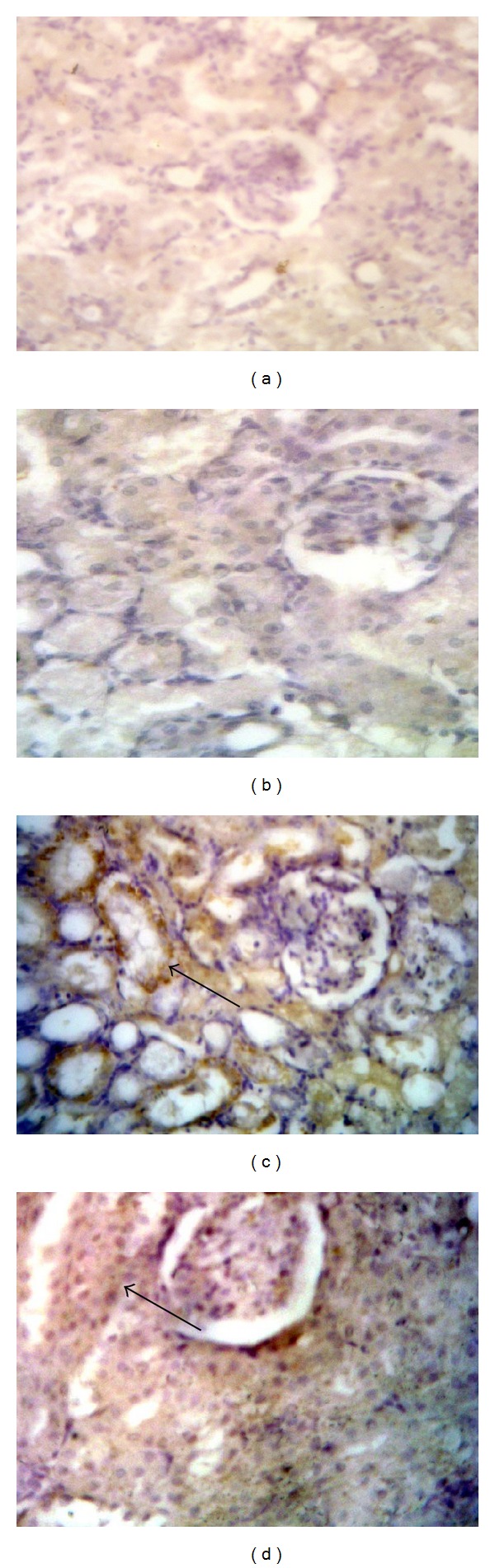
Effect of sildenafil on inducible nitric oxide synthase (iNOS) immunohistochemical staining of gentamicin-treated rat kidney (×400). Localization of iNOS immunoreactivity in the kidney cortex of (a) and (b) control and sildenafil treated groups, respectively, showing negative immunoreactivity, (c) gentamicin-treated group showing high cytoplasmic expression within the renal tubules (arrow), and (d) gentamicin and sildenafil treated group showing faint cytoplasmic expression within few renal tubules (arrow).

**Figure 4:**
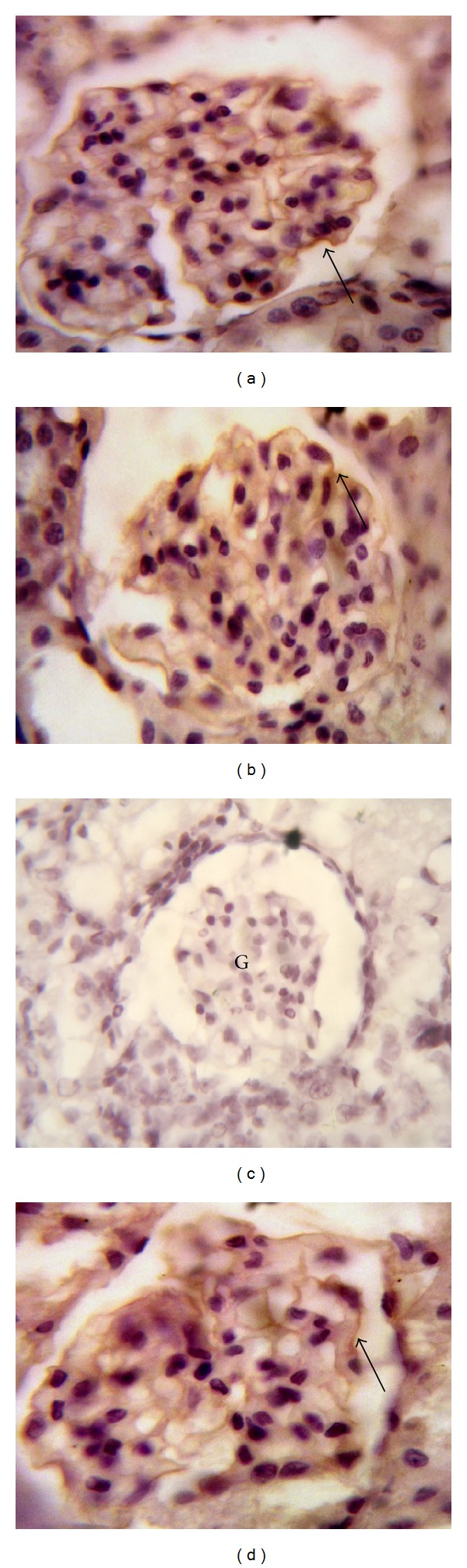
Effect of sildenafil on endothelial nitric oxide synthase (eNOS) immunohistochemical staining of gentamicin-treated rat kidney (×1000). Localization of eNOS immunoreactivity in the kidney cortex of (a) and (b) control and sildenafil-treated groups, respectively, showing positive immunoreactivity in the glomerulus endothelium (arrow), (c) gentamicin-treated group showing absent immunoreactivity within the renal glomerulus (G), and (d) gentamicin and sildenafil-treated group showing immunoreactivity within the renal glomerulus (arrow).

Immunohistochemical staining of rat kidney showed that administration of gentamicin caused significant increase in the immunoreactivity of iNOS and decrease in the immunoreactivity of eNOS compared to control group. Concomitant administration of sildenafil with gentamicin significantly decreased iNOS expression while expression of eNOS was significantly increased compared to gentamicin group (Figures 3 and 4).

## 4. Discussion

The nephrotoxicity of the aminoglycoside antibiotic gentamicin is well documented [1]. In the present study, sildenafil ameliorated gentamicin-induced nephrotoxicity as it reduced serum urea and creatinine as well as urinary albumin levels and restored the histological pattern. In accordance with these results, Choi et al. [8] reported that sildenafil reduced blood urea nitrogen and serum creatinine levels and attenuated renal tubular injuries in renal ischemic reperfusion injury in rats.

It is known that oxidative stress plays an essential role in the development of gentamicin nephrotoxicity. In the present study, the ability of sildenafil to ameliorate gentamicin-induced elevation in malondialdehyde level is consistent with the findings of Cadirci et al. [7] who showed that sildenafil decreases malondialdehyde in the kidney tissues of cecal ligation and puncture-induced septic rats. Moreover, sildenafil treatment decreased MDA levels in renal ischemia-reperfusion injury in rats [14]. This inhibitory effect of sildenafil on lipid peroxidation may be a result of its suppressing effect on iNOS expression [15]. Alternatively, in harmony with the present data, several studies denoted similar findings regarding the ability of sildenafil to increase catalase [10, 16, 17] and superoxide dismutase [7, 17] activities.

In the current study, gentamicin-treated group increased renal nitrite/nitrate levels. The gasotransmitter NO is important in many physiological and pathological processes in kidney. Physiologically, NO is important not only in the regulation of renal hemodynamics but also in the regulation of renal tubular function [18]. However, Narita et al. [19] reported that limiting NO production decreases glomerular injury and subsequent glomerulosclerosis. In agreement with the present study, Christo et al. [3] reported that NO has a role in the acute renal failure caused by gentamicin because the free radical nature of NO might contribute to tubular damage. In addition, NO increases renal injury through its reaction with superoxide radical and generation of a cytotoxic peroxynitrite [20], which could damage the tubular cells resulting in renal failure. On the other hand, the decreased renal NO content in sildenafil-treated gentamicin group is in accord with Yildiz et al. [21] who reported that sildenafil was able to prevent the increase in NO level induced by testicular torsion. In the same line, Zhao et al. [22] reported that sildenafil effectively inhibited lipopolysaccharide-induced production of NO both in N9 cells and primary rat microglial cells. The decrease in NO level may be due to decrease in iNOS level although eNOS level is increased as the amount of NO generated by eNOS is small while large quantities of NO are synthesized by iNOS [23].

The effect of sildenafil on iNOS level is controversial. On one hand, sildenafil increased iNOS expression in ischemia-reperfusion renal injury in rats [8]. On the other hand, Lee et al. [24] found that administration of sildenafil upregulated the hepatic protein expression of phospho-eNOS without enhancing iNOS expression. Alternatively, in line with the present study, the decreased iNOS level in sildenafil-treated gentamicin group is supported by several studies [10, 14, 25, 26]. On the other hand, in the present study, the increased renal eNOS content in sildenafil-treated gentamicin group is in line with previous studies [8, 10, 27]. Collectively, in accord with the current study, Furusu et al. [28] found that the extent of eNOS expression is negatively correlated with the degree of glomerular injury, while the extent of iNOS expression is positively correlated with the degree of glomerular injury in the same tissues. Moreover, transient eNOS-mediated NO production is essential for vasorelaxation, antiapoptosis, and protection against oxidative stress while sustained iNOS-mediated NO generation may mediate lipid peroxidation, DNA damage, and proapoptotic effects [15, 29].

## 5. Conclusions

In conclusion, sildenafil treatment attenuates gentamicin nephrotoxicity in rats partly through ameliorating oxidative stress by preserving the activity of catalase and superoxide dismutase as well as inhibiting iNOS expression and inducing eNOS production.

## Figures and Tables

**Table 1:** Effect of sildenafil on serum creatinine and urea as well as urinary albumin levels of gentamicin-induced nephrotoxicity in rats.

Group	Creatinine mg/dL	Urea mg/dL	Albumin mg/dL
Control	0.53 ± 0.01	26.2 ± 0.24	0.36 ± 0.01
Sildenafil	0.55 ± 0.01	25.8 ± 0.17	0.34 ± 0.01
Gentamicin	3.1 ± 0.1^∗°^	139 ± 1.48^∗°^	4.05 ± 0.10^∗°^
Gentamicin + sildenafil	0.94 ± 0.01^∗°×^	59.6 ± 1.02^∗°×^	2.04 ± 0.06^∗°×^

Data are mean ± SEM of 6–8 rats. ^∗,°,×^Significantly different from control, sildenafil, and gentamicin groups, respectively, at *P* < 0.05.
